# The effects of flutamide on the neonatal rat hypothalamic–pituitary–adrenal and gonadal axes in response to hypoxia

**DOI:** 10.14814/phy2.14318

**Published:** 2019-12-25

**Authors:** Santiago Rolon, Christine Huynh, Maya Guenther, Minhal Gardezi, Jonathan Phillips, Ashley L. Gehrand, Hershel Raff

**Affiliations:** ^1^ Endocrine Research Laboratory Aurora St. Luke’s Medical Center Aurora Research Institute Milwaukee Wisconsin; ^2^ Department of Medicine Medical College of Wisconsin Milwaukee Wisconsin; ^3^ Department of Surgery Medical College of Wisconsin Milwaukee Wisconsin; ^4^ Department of Physiology Medical College of Wisconsin Milwaukee Wisconsin

**Keywords:** ACTH, corticosterone, estradiol, gonadotropins, testosterone

## Abstract

Hypoxia is common with preterm birth and may lead to long‐term effects on the adult hypothalamic–pituitary–adrenal (HPA) axis that are sexually dimorphic due to neonatal androgens. Although the adult rat adrenal does not express appreciable CYP17 activity, the neonatal rat adrenal may synthesize androgens that could be a critical local factor in the development of adrenal function. We evaluated these phenomena by pretreating the neonatal rats on postnatal days (PD) 1, 6, 13, 20 with flutamide (a nonsteroidal androgen receptor antagonist) at a standard or a high dose (10 mg/kg or 50 mg/kg) compared to vehicle control. One day later, neonatal rats were exposed to acute hypoxia and blood was sampled. We found that (a) in PD2 pups, flutamide augmented corticosterone responses in a sexually dimorphic pattern and without an increase in ACTH, (b) PD7 and PD14 pups had the smallest corticosterone response to hypoxia (c) PD21 pups had an adult‐like corticosterone response to hypoxia that was sexually dimorphic, (d) flutamide attenuated ACTH responses in PD7 hypoxic pups, and (e) high‐dose flutamide suppressed the HPA axis, FSH, and estradiol. Flutamide demonstrated mixed antagonist and agonist effects that changed during the first three weeks of neonatal life. We conclude that the use of flutamide in neonatal rats to evaluate androgen‐induced programming of subsequent adult behavior is not optimal. However, our studies suggest neonatal androgens play a role in regulation of adrenal function that is sexually dimorphic and changes during early development.

## Graphical abstract

Hypoxia is common with preterm birth and may lead to long‐term effects on the adult hypothalamic‐pituitary‐adrenal (HPA) axis that are sexually dimorphic due to neonatal androgens. We evaluated this by pretreating the neonatal rats on postnatal days (PD) 1, 6, 13, 20 with flutamide (a non‐steroidal androgen receptor antagonist); 1 day later, neonatal rats were exposed to acute hypoxia and blood was sampled. We conclude that the use of flutamide in neonatal rats to evaluate androgen‐induced programming of subsequent adult behavior is not optimal and that neonatal androgens play a role in regulation of adrenal function that is sexually dimorphic and changes during early development.

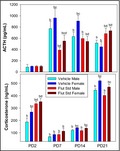

## 1 INTRODUCTION

Common neonatal stressors like hypoxia may affect the subsequent behavior of the adult hypothalamic–pituitary–adrenal (HPA) axis in a sexually dimorphic manner (Gehrand et al., 2018; Seale, Wood, Atkinson, Bate, et al., 2004; Seale, Wood, Atkinson, Harbuz, & Lightman, 2004). We previously found that neonatal stressors like hypoxia and maternal separation program the control of the HPA axis, insulin and glucose dynamics, and other metabolic biomarkers in the adult rat in a sexually dimorphic manner (Gehrand et al., 2018, 2016; Raff et al., 2018). Previous studies have shown that stress‐induced testosterone may be responsible for long‐term, sexually dimorphic programming in male rats (Brunton, 2015; Jopek et al., 2017; Kasprzak & Lesniewska, 1986; Kitay, 1963a, 1963b; Seale, Wood, Atkinson, Harbuz, et al., 2004; Seale, Wood, Atkinson, Lightman, & Harbuz, 2005).

Flutamide (Eulexin^®^) is a clinically used nonsteroidal androgen receptor (AR) antagonist in adults. However, flutamide can act like an agonist at high doses due to stabilization of the AR (Kemppainen & Wilson, 1996; Nguyen, Yao, & Pike, 2007). Although the adult rat adrenal does not express CYP17 and cannot synthesize appreciable androgens (Gallo‐Payet & Battista, 2014), the neonatal rat adrenal may have the capacity to synthesize androgens that could be a critical local factor in the development of normal adrenal function (Pignatelli, Xiao, Gouveia, Ferreira, & Vinson, 2006; Zhou, Kang, Chen, Han, & Ma, 2015). Furthermore, the neonatal adrenal may express the AR making it susceptible to circulating testosterone from the gonads (Brownie, Colby, Gallant, & Skelton, 1970; Calandra et al., 1978; Rifka, Cutler, Sauer, & Loriaux, 1978).

The purpose of this study was to assess the effectiveness and consistency of flutamide, an AR modulator, as a useful antagonist in the neonatal rat. If flutamide does not have reliable AR antagonist activity from birth to weaning, it may not be useful to evaluate the role of androgens in the programming by neonatal stressors on subsequent adult sexually‐dimorphic behavior. Therefore, we evaluated the effect of standard and high‐dose flutamide pretreatment on the neonatal ACTH, corticosterone, LH, FSH, testosterone (in males) and estradiol (in females) responses to hypoxia—a common neonatal stressor.

## 2 MATERIALS AND METHODS

### 2.1 Animal treatment and experimental protocols

Timed‐pregnant Sprague‐Dawley (*SD*) female rats (*N* = 75) were obtained from Envigo, maintained in a controlled environment (lights on 06:00 to 18:00 hr), and given a standard diet and water ad libitum (Gehrand et al., 2016; Gehrand, Phillips, Malott, & Raff, 2019). Dams delivered pups spontaneously and cared for rat pups without interruption until experimentation. Federal guidelines (https://grants.nih.gov/grants/olaw/references/phspol.htm) were followed for use and handling of laboratory animals. Protocols were approved by the Institutional Animal Care and Use Committee of Aurora Health Care.

### 2.2 Age groups of rat pups

Rat pups of both sexes were studied on four postnatal days (PD) in order to account for the age‐dependent changes in HPA‐axis stress response within the neonatal period (Bruder, Kamer, Guenther, & Raff, 2011; Bruder, Taylor, Kamer, & Raff, 2008). On PD1, PD6, PD13, or PD20, male and female rats were randomly divided into flutamide or vehicle treatment (as described below) and then studied 24 hr later (on PD2, PD7, PD14, or PD21).

#### 2.2.1 Drug treatment groups (PD1, PD6, PD13, PD20)

Flutamide (Sigma‐Aldrich, F9397) or vehicle (sesame oil; Sigma‐Aldrich, S3547, St. Louis, MO) was given subcutaneously 24 hr before experimentation. We chose 24 hr pretreatment because it is required for the full effect of flutamide in the perinatal rat (Lieberburg, MacLusky, & McEwen, 1980). After injection, pups were maintained with their original litters and returned to their lactating dams.

##### Flutamide ‐standard dose or vehicle

Male and female rat pups were administered 10 mg/kg flutamide or vehicle control (10 µl/g body weight sesame oil) subcutaneously. The 10 mg/kg dosage was chosen because it achieves effective androgen blockade without marked effects on sex organ weight, gonadal development, and rat sexual characteristics (Gray et al., 2006). As previously validated, 10 mg/kg of flutamide dosage modulates the AR in the neonatal rat with minimal hepatotoxicity (McCormick & Mahoney, 1999). Furthermore, 10 mg/kg flutamide produces significant AR modulation within the neonatal rat HPA axis without a significant effect on anogenital distance and early‐life body weight (McCormick & Mahoney, 1999).

##### Flutamide ‐high dose

After analyzing the results of experiments described above, additional groups of male and female rat pups were given a higher dose of flutamide (50 mg/kg subcutaneously) to confirm that our findings with the lower dose were not due to under‐dosing. A high dosage was not initially chosen for experimentation because previous studies have shown that higher doses result in decreased sex organ weight, nipple retention, hypospadias, reduced penile length, reduced anogenital distance and late pubertal development (Gray et al., 2006).

#### 2.2.2 Experiments (PD 2, 7, 14, 21)

Twenty‐four hours after flutamide injection, male and female pups within each drug pretreatment group were separated from their lactating dams and randomized into the three experimental groups as described below. *N* values for each result are listed in the Table and Figure legends. Trunk blood samples were collected in tubes containing K_2_EDTA. Blood from 2–3 PD2 pups were pooled to achieve an adequate plasma sample volume for all measurements as we described previously (Bruder et al., 2011). Each pooled sample was considered *n* = 1 for the purposes of statistical analysis. PD7, PD14 and P21 pup samples consisted of one pup per K_2_EDTA tube.

##### Baseline

Pups were removed from their home cage with lactating dams and trunk blood samples were immediately obtained by decapitation.

##### Normoxia (time control for hypoxia) with maternal separation

Pups were separated from their dams and placed into an environmental chamber (vented with room air) for 90 min. PD2 and PD8 pups were placed on low‐heated bedding within the chamber as described previously to prevent hypothermia associated with maternal separation alone in younger neonatal rat pups (Gehrand et al., 2016).

##### Hypoxia with maternal separation

Pups were separated from their lactating dams and placed in the environmental chamber as per the normoxic treatment including the low‐heated bedding. Hypoxia was achieved by decreasing inspired oxygen to 8% O_2_ for 90 min (Bruder et al., 2011, 2008; Guenther, Bruder, & Raff, 2012). We chose 90 min of normoxia (described above) and hypoxia because (a) the phenomena we have studied up to now plateaus between 60 and 120 min (Bruder et al., 2008), (b) 90 min of separation is consistent with normal, spontaneous rat behavior (Calhoun, 1963) (c) longer periods of separation result in hypoinsulinemia, which we wanted to avoid (Guenther et al., 2012).

At the end of 90 min of normoxia or hypoxia, trunk blood samples were obtained by decapitation.

### 2.3 Plasma hormone assays

Plasma ACTH and corticosterone were measured by radioimmunoassay as described and validated previously (Raff, Hong, Oaks, & Widmaier, 2003). Plasma samples were analyzed for FSH and LH at the Ligand Assay and Analysis Core within the Center for Research in Reproduction at the University of Virginia (UVA) School of Medicine by multiplex testing (EMD Millipore). Values lower than 0.2 ng/ml (the FSH/LH assay sensitivity) were assigned a value of 0.2 ng/ml and were not used in further statistical analysis if the majority of the results were below 0.2 ng/ml. Male LH samples were not subject to statistical analysis since most of the results were below the limit of detection. Plasma testosterone levels in male pups were measured by LC‐MS/MS as described previously (Raff et al., 2018). Plasma estradiol levels in female pups were measured via a validated ELISA (Calbiotech #ES180S‐100) as described previously (Haisenleder, Schoenfelder, Marcinko, Geddis, & Marshall, 2011).

### 2.4 Statistical analyses

Data were analyzed by three‐way ANOVA without and with log_10_ transformation and Duncan's multiple range test (SigmaPlot 12.5; Systat Software, Inc.). The comparisons of the magnitude of the responses to hypoxia *between* age groups were not formally analyzed because age‐dependent differences regarding the neonatal rat HPA axis stress response has been previously described (Walker, Perrin, Vale, & Rivier, 1986; Walker, Scribner, Cascio, & Dallman, 1991) and the between age data comparisons were heteroscedastic even after log_10_ transformation. One‐ and 2‐way ANOVA, Mann–Whitney Rank Sum Test, and *t*‐tests were occasionally employed only to explore those complete data sets with high residual variance by 3‐way ANOVA and are so identified where appropriate in the text. *p* < .05 was considered statistically significant. Values are reported as mean ± *SE*.

## 3 RESULTS

### 3.1 ACTH and corticosterone

#### 3.1.1 Baseline and normoxia separation (Table 1)

In PD2 pups, there were no significant differences in plasma ACTH or corticosterone between flutamide treatments or sex, although corticosterone tended to increase with flutamide (either dose) (Table 1). There was no statistically significant effect of 90 min of normoxic separation (compared to baseline) on plasma ACTH. Plasma corticosterone was significantly lower than baseline in the pups treated with the standard dose of flutamide.

Like the PD2 pups, there were no significant effects of flutamide on baseline plasma ACTH and corticosterone in PD7 pups (Table 1). As opposed to the PD2 pups, corticosterone increased in the PD7 vehicle and standard dose flutamide pups after 90 min of normoxic separation compared to baseline. This effect was not observed with high‐dose flutamide. In the high‐dose flutamide pups, plasma ACTH and corticosterone were significantly lower after normoxic separation compared to the standard dose flutamide pups.

In PD14 pups, 90 min of normoxic separation led to an increase in plasma ACTH and corticosterone in all treatment groups and both sexes (Table 1). The concentrations of plasma ACTH and corticosterone in PD21 pups overall were quite variable. The major finding was that the standard dose of flutamide led to a lower plasma corticosterone level after 90 min of normoxic separation in the male compared to the female rat pups.

The major differences between age groups are presented in Table 1 to set the stage for the description of the responses to hypoxia shown below. Overall, as described previously (Wood & Walker, 2015) the plasma ACTH concentrations were higher at PD14 and PD21 compared to PD7. PD21 pups had the highest plasma corticosterone concentrations approaching levels achieved in rats as adults (Wood & Walker, 2015).

**Table 1:** Effect of flutamide pretreatment (standard [10 mg/kg] or high dose [HD; 50 mg/kg]) and vehicle on plasma ACTH (pg/ml) and corticosterone (ng/ml) at baseline and after 90 min of normoxic separation at different ages

Age	Treatment/Sex	Baseline		Normoxic Separation (90 min)	
		ACTH	Corticosterone	ACTH	Corticosterone
PD2	Vehicle/Male	112 (21)	44.9 (8.5)	76 (4)	34.6 (3.4)^w^
	Vehicle/Female	87 (15)	48.7 (6.0)	82 (12)	55.2 (7.3)^w^
	Flutamide/Male	103 (15)	65.8 (8.4)	86 (10)	34.8 (5.0)^aw^
	Flutamide/Female	105 (13)	60.6 (4.8)	67 (8)	37.8 (10.0)^ aw^
	HD Flutamide/Male	80 (8)	58.9 (13.1)	59 (8)	42.9 (3.5)^w^
	HD Flutamide/Female	75 (5)	64.7 (11.6)	56 (7)	45.1 (7.2)^w^
PD7	Vehicle/Male	52 (4)^z^	8.0 (0.6)^z^	69 (9)	27.0 (7.0)^ aw^
	Vehicle/Female	52 (4)^z^	7.0 (1.0)^z^	71 (4)	22.0 (3.0)^ aw^
	Flutamide/Male	45 (5)^z^	13.0 (4.0)^z^	75 (5)	34.0 (6.0)^ aw^
	Flutamide/Female	52 (5)^z^	10.0 (1.0)^z^	75 (4)	23.0 (7.0)^ aw^
	HD Flutamide/Male	58 (7)	15.3 (6.7)	51 (4)^v^	15.6 (2.8)^v^
	HD Flutamide/Female	53 (4)	5.3 (1.1)	59 (5)^v^	12.8 (2.3)^v^
PD14	Vehicle/Male	55 (4)^z^	17.0 (3.0)^z^	150 (40)^az^	51.0 (7.0)^awx^
	Vehicle/Female	68 (9)^z^	24.0 (12.0)^z^	201 (56)^az^	71.0 (6.0)^awx^
	Flutamide/Male	56 (4)^z^	15.0 (1.0)^z^	154 (29)^az^	47.0 (6.0)^awx^
	Flutamide/Female	58 (7)^z^	16.0 (2.0)^z^	111 (21)^az^	57.0 (7.0)^awx^
	HD Flutamide/Male	67 (4)	21.6 (5.8)	97 (7)^az^	51.5 (8.6)^awx^
	HD Flutamide/Female	63 (6)	29.6 (8.l)	142 (29)^az^	78.7 (8.9)^awz^
PD21	Vehicle/Male	58 (5)^y^	107.0 (5.0)^yz^	113 (21)^xz^	151.0 (37.0)^a^
	Vehicle/Female	99 (21)^y^	168.0 (20.0)^cyz^	105 (18)^xz^	153.0 (32.0)
	Flutamide/Male	73 (11)^y^	86.0 (20.0)^yz^	119 (25)^xz^	165.0 (38.0)^a^
	Flutamide/Female	103 (27)^y^	110.0 (22.0)^cyz^	146 (32)^xz^	236.0 (48.0)^ac^
	HD Flutamide/Male	126 (40)	211.7 (55.3)^v^	116 (28)	95.9 (30.2)
	HD Flutamide/Female	123 (18)	178.2 (58.7)	92 (13)	139.0 (59.7)

Note

Data are shown as mean (*SEM*). *N* values are PD2 (Pooled Samples) Vehicle/Male (8–10), Vehicle/Female (10–11), Flutamide/Male (10–12), Flutamide/Female (11–14), High Dose (HD) Flutamide (2–4); PD7 Vehicle/Male (9–11), Vehicle/Female (8–9), Flutamide/Male (10–12), Flutamide/Female (10–12), HD (3–8); PD14 Vehicle/Male (9–12), Vehicle/Female (10–11), Flutamide/Male (13), Flutamide/Female (10–13), HD (8); PD21 Vehicle/Male (8–9), Vehicle/Female (8–9), Flutamide/Male (7–9), Flutamide/Female (10), HD (4–6).

^a^different from baseline; ^c^different within sex, ^z^age group different from PD2 within column. ^y^different from PD7 and PD14 within column. ^x^Different from PD7 within column. ^w^different from PD21 within column, ^v^different from standard dose within sex. All *p* < .05.

#### 3.1.2 Responses to hypoxia

**Figure 1:**
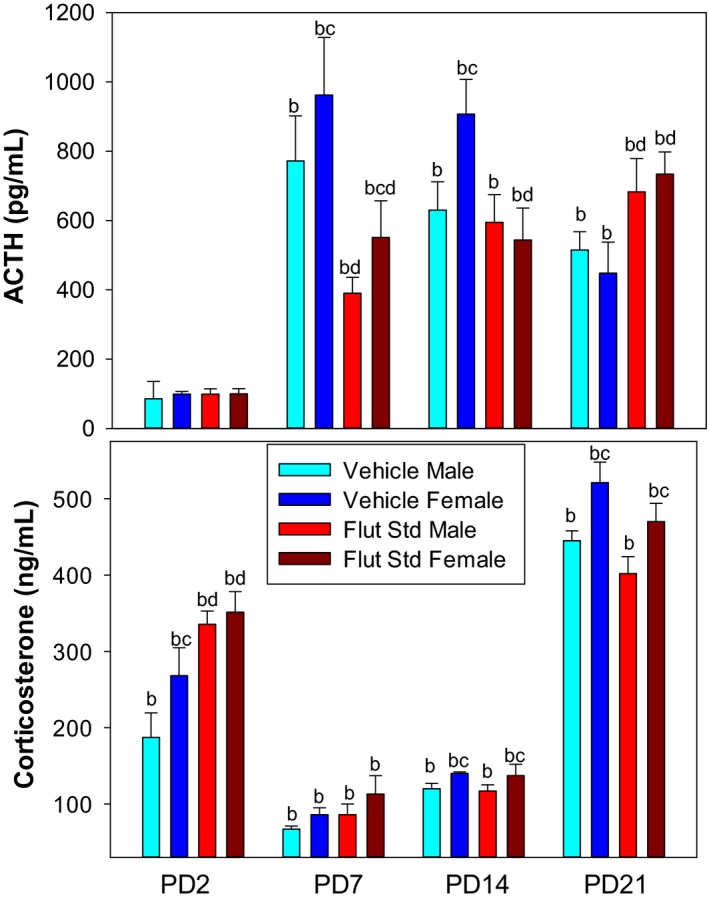
Plasma ACTH and corticosterone responses to hypoxia in PD2, PD7, PD14, PD21 pups after pretreatment with vehicle or standard‐dose flutamide (10 mg/kg). Values are mean ± *SE*. *N* values are PD2 (Pooled Samples) Vehicle/Male (8–10), Vehicle/Female (10–11), Flutamide/Male (10–12), Flutamide/Female (11–14); PD7 Vehicle/Male (9–11), Vehicle/Female (8–9), Flutamide/Male (10–12), Flutamide/Female (10–12); PD14 Vehicle/Male (9–12), Vehicle/Female (10–11), Flutamide/Male (13), Flutamide/Female (10–13); PD21 Vehicle/Male (8–9), Vehicle/Female (8–9), Flutamide/Male (7–9), Flutamide/Female (10). ^b^different from normoxia (see Table 1 for normoxia data); ^c^different within sex; ^d^effect of flutamide within age

Figure 1 shows the effect of the standard dose of flutamide (10 mg/kg) on the plasma ACTH and corticosterone responses to hypoxia. Because of heteroscedasticity of the data, we are only presenting the within age comparisons since that is the focus of the hypothesis addressed in this study and because the between age differences have been described previously (Bodager, Gessert, Bruder, Gehrand, & Raff, 2014; Wood & Walker, 2015).

PD2 rat pups showed a large and significant increase in corticosterone with no change in immunoreactive ACTH confirming what we have shown previously (Gehrand et al., 2019). The corticosterone response to hypoxia was greater in vehicle‐treated PD2 females compared to males. Flutamide treatment resulted in an augmentation in the corticosterone response to hypoxia in both female and male PD2 pups.

There was a significant increase in ACTH in PD7, PD14, and PD21 rat pups compared to baseline. Flutamide resulted in decreased concentrations of ACTH in PD7 pups of both sexes. Flutamide resulted in significantly decreased concentrations of ACTH compared to vehicle in female PD14 pups only. This drug effect, however, did not result in different corticosterone concentrations. Vehicle‐treated PD7 and PD14 females showed greater ACTH responses to hypoxia compared to males. While PD7 pups showed no sex difference in corticosterone response to hypoxia, PD14 females showed greater concentrations of corticosterone compared to males. Overall PD21 pups paralleled the stress response observed in the adult rat with large ACTH and corticosterone responses to hypoxia (Wood & Walker, 2015). Flutamide pretreatment in PD21 pups resulted in higher concentrations of ACTH compared to vehicle pretreatment. Vehicle and flutamide‐pretreated female PD21 pups showed a greater corticosterone response to hypoxia in both treatments.

**Figure 2:**
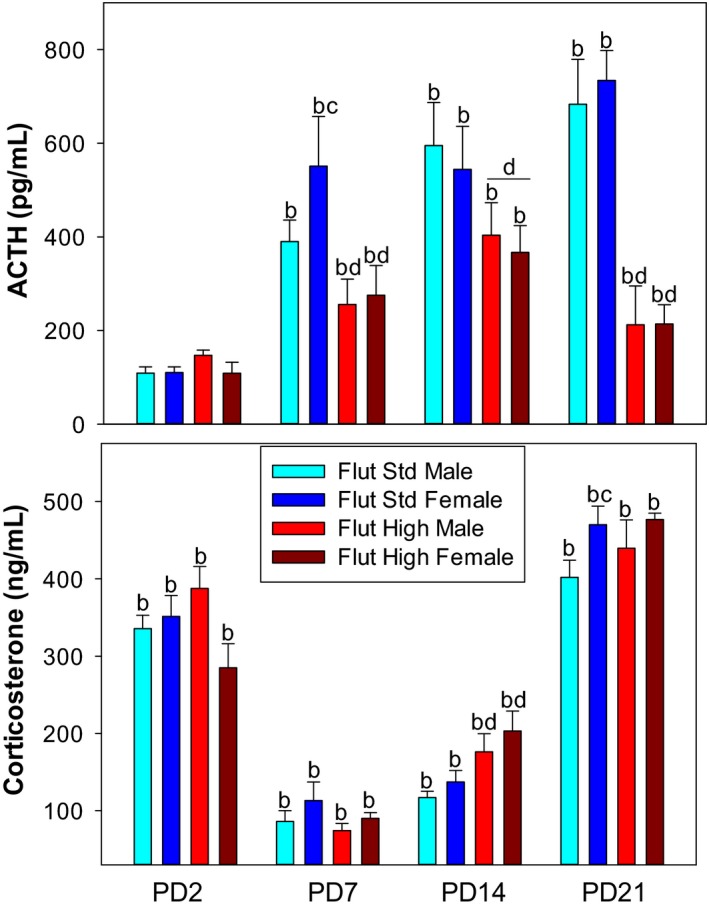
Plasma ACTH and corticosterone responses to hypoxia in PD2, PD7, PD14, PD21 rats after pretreatment with standard dose flutamide (10 mg/kg) or high‐dose flutamide (50 mg/kg). Values are mean ± *SE*. *N* values are PD2 (Pooled Samples) Flutamide/Male (10–12), Flutamide/Female (11–14), High–Dose (HD) Flutamide (2–4); PD7 Flutamide/Male (10–12), Flutamide/Female (10–12), HD (3–8); PD14 Flutamide/Male (13), Flutamide/Female (10–13), HD (8); PD21 Flutamide/Male (7–9), Flutamide/Female (10), HD (4–6).^ b^different from normoxia (see Table 1 for normoxia data); ^c^different within sex; ^d^effect of flutamide within age

Figure 2 compares the effect of the standard dose of flutamide (10 mg/kg) and high‐dose flutamide (50 mg/kg) on the plasma ACTH and corticosterone responses to hypoxia. The higher flutamide dosage was used to confirm that the results in Figure 1 were not due to an inadequate dosage. Since our objective was to characterize the effect of flutamide within each neonatal developmental age, we were interested in mainly describing the effect of hypoxia within each age group. Consequently, we are presenting the within age group statistical comparisons and focusing on major differences between the two doses of flutamide.

There was no significant difference in plasma ACTH or corticosterone between flutamide doses in PD2 pups. High‐dose flutamide in PD7, PD14, and PD21 pups resulted in an attenuated ACTH plasma response to hypoxia compared to standard dose flutamide. However, PD7 corticosterone plasma concentrations were not significantly different between high and standard dose flutamide treatments. It is important to note that PD7 adrenal glands are stress and ACTH hyporesponsive (Bodager et al., 2014; Bruder et al., 2008; Chintamaneni, Bruder, & Raff, 2013; Zilz, Li, Castello, Papadopoulos, & Widmaier, 1999). High‐dose flutamide in PD14 pups showed a significant increase in the plasma corticosterone response to hypoxia; however, this effect was not present in the PD21 pups. It is important to note that the corticosterone responses to hypoxia in PD21 pups is probably at the adrenocortical steroidogenic maximum for that age (Wood & Walker, 2015). As described in Figure 1, female PD7 pups treated with standard dose flutamide had an augmented ACTH response and PD21 had an augmented corticosterone response to hypoxia compared to male pups. This sexual dimorphism was not observed in pups treated with high‐dose flutamide.

### 3.2 FSH, LH, testosterone, and estradiol

**Figure 3:**
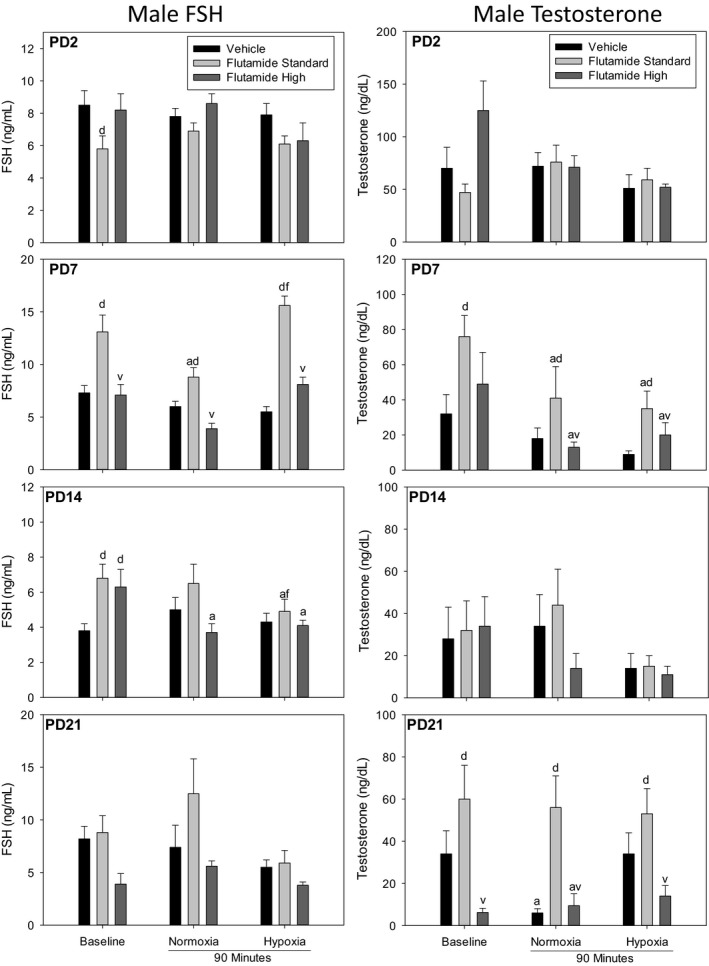
Male plasma FSH and testosterone concentrations after pretreatment with vehicle, standard dose flutamide (10 mg/kg) or high‐dose flutamide (50 mg/kg) in samples taken at baseline, after normoxic time control, and in response to hypoxia. Values are mean ± *SE*. *N* values are PD2 (Pooled Samples) Vehicle (8–10), Flutamide (10–12), HD (2–4); PD7 Vehicle (9–11), Flutamide (10–12), HD (3–8); PD14 Vehicle (9–12), Flutamide (13), HD (8); PD21 Vehicle (8–9), Flutamide (7–9), HD (4–6). ^a^different from baseline; ^d^effect of flutamide within age; ^f^hypoxia different from normoxia; ^v^different from standard dose within sex

Figure 3 describes the effects of flutamide pretreatment on baseline, normoxic time control, and hypoxia on male neonatal FSH and testosterone. As before, only the significant within‐age differences are described.

Overall, the only significant difference observed in the PD2 male response was a decrease in FSH plasma concentration after treatment with standard dose flutamide, under baseline conditions. There was a tendency for the higher dose of flutamide to increase baseline testosterone in PD2 pups. In the PD7 male, baseline, normoxic time control, and hypoxic pups pretreated with the standard flutamide dose showed increased plasma FSH and testosterone compared to vehicle. However, this effect was lost with high‐dose flutamide treatment. In the PD14 male pups, both standard and high‐dose flutamide resulted in increased baseline FSH. In the PD21 male pups, standard dose flutamide resulted in significantly increased testosterone concentrations; high‐dose flutamide significantly lowered baseline and normoxia controls as well as hypoxia‐stimulated plasma testosterone concentrations.

**Figure 4:**
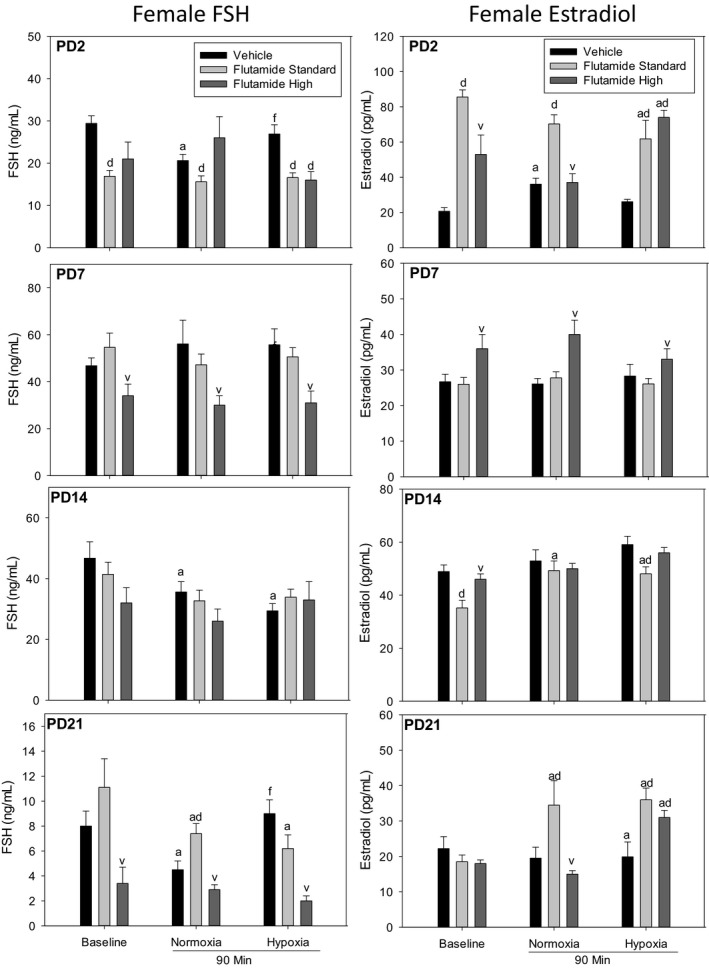
Female FSH and estradiol after pretreatment of PD2, PD7, PD14, PD21 rats with vehicle, standard dose flutamide (10 mg/kg) or high‐dose flutamide (50 mg/kg) in samples taken at baseline, after normoxic time control, and in response to hypoxia. Values are mean ± *SE*. *N* values are PD2 (Pooled Samples) Vehicle (10–11), Flutamide (11–14), HD (2–4); PD7 Vehicle (8–9), Flutamide (10–12), HD (3–8); PD14 Vehicle (10–11), Flutamide (10–13), HD (8); PD21 Vehicle (8–9), Flutamide (10), HD (4–6). ^a^different from baseline; ^d^effect of flutamide within age; ^v^different from standard dose within sex

Figure 4 describes the within‐group, age‐specific differences in the female pituitary–gonadal responses to parallel the previous paragraph concerning the data in males. In the PD2 female, standard dose flutamide decreased FSH and increased estradiol plasma concentrations. A similar effect was observed with high‐dose flutamide, but only in female pups under hypoxia. In the PD7 female, high‐dose flutamide decreased FSH and increased estradiol concentrations. PD14 females treated with standard dose flutamide had lower plasma estradiol concentrations at baseline and with hypoxia compared to vehicle.

In the PD21 female, high‐dose flutamide decreased FSH plasma concentrations. A greater estradiol response to normoxia occurred with standard dose flutamide pretreatment compared to vehicle and high‐dose flutamide. Standard and high‐dose flutamide pretreatment led to an increase in estradiol in response to hypoxia (compared to baseline) that was not observed in the vehicle treated PD21 pups. Finally, although we have described the within‐group variations in the pituitary–gonadal response to hypoxia, it is important to point out that, overall, FSH in the PD21 rat pups appeared to be lower than in other age groups.

Table 2 shows LH concentrations in female pups after pretreatment with the standard dose of flutamide. Male LH data are not shown because more than 50% of results were below the limit of detection of the assay. Since there were no significant differences between high and standard dose flutamide with respect to LH, only the standard dose is shown. In order to maintain perspicuity, data presented below are divided into within and between‐group interactions. In PD2 females, LH decreased to the same extent with normoxia and hypoxia. An effect of drug treatment was only observed under baseline conditions, resulting in lower LH plasma concentrations. In the PD7 female, hypoxia induced increases in plasma LH concentrations in the vehicle and flutamide pretreatment groups compared to baseline. In PD14, normoxia and hypoxia resulted in a decrease in LH plasma concentrations. Under normoxic conditions, flutamide pretreatment resulted in higher LH concentrations compared to vehicle. Under hypoxic conditions, LH was significantly lower compared to baseline and there was no difference between flutamide and vehicle pretreatment. In the PD21 female, flutamide pretreatment increased baseline plasma LH; normoxia or hypoxia decreased plasma LH from these increased baseline concentrations.

**Table 2:** Plasma LH (ng/ml) in female pups pretreated (24 hr before) with the standard dose of flutamide (10 mg/kg)

Age	Treatment	Baseline	Separation (90 min)	
			Normoxia	Hypoxia
PD2	Vehicle	2.8 (0.4)	0.8 (0.2)^ax^	1.9 (0.7)^ax^
	Flutamide	1.5 (0.3)^d^	1.0 (0.2)	1.3 (0.2)^x^
PD7	Vehicle	2.1 (0.5)	3.6 (1.0)	5.7 (1.3)^a ^
	Flutamide	2.3 (1.1)	1.5 (0.4)	4.2 (1.0)^ab^
PD14	Vehicle	6.3 (2.9)	0.8 (0.3)^ax^	0.6 (0.1)^ax^
	Flutamide	6.4 (2.1)^z^	3.5 (2.7)^ad^	0.8 (0.1)^abx^
PD21	Vehicle	0.6 (0.2)	0.3 (0.0)^x^	0.4 (0.1)^x^
	Flutamide	2.3 (1.0)^d^	0.3 (0.0)^a^	0.4 (0.0)^ax^

Note

Data are shown as mean (*SEM*). *N* values are PD2 (Pooled Samples) Vehicle (10–11), Flutamide (11–14), PD7 Vehicle (8–9), Flutamide (10–12), PD14 Vehicle (10–11), Flutamide (11–13), PD 21 Vehicle (8–9), Flutamide (10). LH for males below detection in more than 50% of the samples so are not shown. High‐dose flutamide had no effect compared to standard‐dose flutamide so is not shown.

^a^different from baseline; ^b^different from normoxia, ^d^effect of flutamide within age, ^z^different from PD2 within treatment. ^x^different from PD7 within treatment.

Overall, PD14 females showed higher baseline LH compared to pups in other age groups both within vehicle and flutamide pretreatments (Table 2). Hypoxia resulted in lower LH concentrations in vehicle pretreated PD2 and vehicle and flutamide pretreated PD14 pups, but increased LH in PD7 in both pretreatment groups. The lowest LH concentrations were observed in PD21 pups within normoxia and hypoxia.

## 4 DISCUSSION

The main motivation for this study was our previous findings that neonatal stressors program the control of HPA axis, insulin and glucose, and other metabolic biomarkers in the adult rat in a sexually dimorphic manner (Gehrand et al., 2018, 2016; Raff et al., 2018). Based on the literature, we thought that stress‐induced testosterone responses in the neonatal male rat could be responsible for this long‐term programming (Boksa & Zhang, 2008; Brunton, 2015; Clarkson & Herbison, 2016; Jopek et al., 2017; Kasprzak & Lesniewska, 1986; Liu & Du, 2002; Slob, Ooms, & Vreeburg, 1980; Vega Matuszczyk, Silverin, & Larsson, 1990; Ward, Ward, Denning, French, & Hendricks, 2002). Flutamide has previously been used in the neonatal rat to modulate the effects of circulating androgens (McCormick & Mahoney, 1999; Ongaro, Castrogiovanni, Giovambattista, Gaillard, & Spinedi, 2011). Before we could utilize flutamide as a reliable, short‐term androgen receptor (AR) antagonist in the neonate to study subsequent adult sexual dimorphisms, we had to establish the dynamics of flutamide action in our early neonatal rat model of prematurity involving maternal separation and hypoxia (Bruder et al., 2011, 2008; Gehrand et al., 2019; Guenther et al., 2012; Johnson, Bruder, & Raff, 2013).

We hypothesized that flutamide, an AR antagonist, would augment the HPA axis response to neonatal hypoxia since androgens typically inhibit the HPA axis and that the magnitude of this effect would vary by developmental neonatal age (Bruder et al., 2008; Chintamaneni et al., 2013). Furthermore, we would expect that AR antagonism would augment the gonadotropin and gonadal steroid response to hypoxia by attenuated gonadal steroid negative feedback (Ba et al., 2000; Kemppainen & Wilson, 1996; McCormick & Mahoney, 1999).

The major findings of this study were (a) PD2 pups treated with vehicle exhibited a large, sexually dimorphic corticosterone response to hypoxia without an increase in plasma ACTH, (b) PD2 exhibited a flutamide‐induced augmentation of corticosterone responses to hypoxia which was not sexually dimorphic, (c) PD7 and PD14 pups exhibited a flutamide‐induced suppression of the ACTH response to hypoxia indicating agonistic activity, (d) PD21 pups pretreated with flutamide had an adult‐like augmentation of the ACTH response to hypoxia indicating the expected antagonist activity, (e) we found the expected increase in FSH and testosterone in males with the standard (but not high) dose of flutamide, (f) PD7 and PD14 females exhibited an LH response to hypoxia without or with flutamide pretreatment suggested an androgen‐receptor independent effect, and (g) PD21 females showed a suppression of FSH with high‐dose flutamide suggesting agonism at the level of the hypothalamus and pituitary.

### 4.1 Sexual dimorphism

We showed that the neonatal rat pups exhibited a large, sexually dimorphic corticosterone response to hypoxia and confirmed that it occurred without an increase in plasma ACTH in PD2 pups (Bruder et al., 2008; Gehrand et al., 2019). We have demonstrated that the ACTH independence in PD2 pups is mediated by a nonimmuno‐assayable form of ACTH (Gehrand et al., 2019). What could be causing the ACTH‐driven sexual dimorphism in the adrenal response to hypoxia? Previous studies have demonstrated that the sexually dimorphic nature of the HPA axis responsiveness is due to early neonatal testosterone (Gorski, Gordon, Shryne, & Southam, 1978). In addition to the direct effect of testosterone on the AR, it is also possible that conversion of androgens to estrogen by aromatase in the rat brain could be responsible for this sexual dimorphism (Bingham, Gray, Sun, & Viau, 2011; Bingham, Williamson, & Viau, 2006). This mechanism may occur through the posterior bed nuclei of the stria terminalis, relaying to the paraventricular nucleus of the hypothalamus, and ultimately mediating the HPA axis response to hypoxia (Bingham, Myung, et al., 2011). Additionally, 3ß‐diol, a metabolite of dihydrotestosterone, may mediate an inhibitory effect on the corticosterone response in male mice by binding to the estrogen receptor (ERß) (Lund, Munson, Haldy, & Handa, 2004). In addition, several sex hormone‐dependent genes such as glutamic acid decarboxylase 65 (GAD65) may be involved in sexual differentiation of the rat brain (Yonehara, Suzuki, Yamanouchi, & Nishihara, 2003). GAD65 is found in the paraventricular nucleus of the hypothalamus and mediates inhibition of the HPA‐axis (Gao, Klomp, Wu, Swaab, & Bao, 2013). This further demonstrates that the sexual‐dimorphic nature of the HPA–axis response to stress is a result of several neuroendocrine interactions that are fully dependent on rat gonadal hormones.

Interestingly, acute hypoxia did not stimulate neonatal testosterone production which does not agree with previous studies (Boksa & Zhang, 2008; Liu & Du, 2002). One possible explanation for this is the prior use of relatively nonspecific immunoassays for testosterone whereas we used a more specific and sophisticated LC‐MS/MS method validated for the use in rat plasma (Raff et al., 2018). Regardless, we showed that flutamide has effects in the nonstressed male neonate confirming that an increase in testosterone is not required to exert important physiological effects (Gray et al., 2006; McCormick, Furey, Child, Sawyer, & Donohue, 1998).

### 4.2 Agonist/Off target effects of flutamide

In the PD2 pups, standard dose flutamide augmented the corticosterone response to hypoxia (confirming antagonistic activity). This effect, however, was not sexually dimorphic. Male pup testosterone may act on the estrogen receptor via its aromatization to estradiol (McEwen, Lieberburg, Chaptal, & Krey, 1977), which could in part explain the lack of sexual dimorphism in some of the responses after pretreatment with flutamide.

Unexpectedly, standard dose flutamide suppressed the ACTH response to hypoxia in male and female PD7 pups. Flutamide demonstrated some agonistic activity which has previously been demonstrated (Lee, 2016b; MacLusky, Hajszan, & Leranth, 2004; Nguyen et al., 2007). This agonist effect may be due to dose‐dependent receptor activation and conformational changes (Wong, Kelce, Sar, & Wilson, 1995). This may result in increased AR transcriptional activity and DNA binding (Wong et al., 1995). Furthermore, flutamide may act as an agonist in the rat brain and mimic the neuroprotective effects of testosterone (Fanaei, Sadeghipour, Karimian, & Hassanzade, 2013). The agonistic actions of flutamide can occur via mechanisms that depend on the AR in both neuronal and non‐neuronal cells (Lee, 2016a, 2016b; MacLusky et al., 2004; Nguyen et al., 2007) as well as other off‐target effects (Foster et al., 2011). Therefore, the suppression of ACTH observed in our study could be the result of AR agonism in the neonatal rat brain. In PD14 pups, flutamide suppressed the ACTH response to hypoxia in female pups only. The PD14 male no longer showed the suppressive effects of flutamide, which seems to suggest that male pup transitions to a PD21‐like (i.e. more mature) state earlier than the female.

PD21 pups had an “adult‐like” augmentation of the ACTH response to hypoxia with standard dose flutamide that was not reflected in the corticosterone response, likely because the adrenal was already at its maximum secretory rate (Arai & Widmaier, 1993; Bodager et al., 2014; Wood & Walker, 2015; Zilz et al., 1999). These high concentrations in plasma corticosterone may be due to a near maximal rate of release of corticosterone into the blood, which is dependent on rat age, adrenal mass, and capillary blood flow (Stockham, 1964). Additionally, the sexual dimorphic corticosterone response to hypoxia in PD21 pups may be due higher CBG levels in the female (Smith & Hammond, 1991). Our observations confirm the findings in other studies showing that CBG levels increase several‐fold during the first two weeks of life in the neonatal rat and that this increase is greater in the female (Johnson et al., 2013).

### 4.3 Stress hyporesponsive period

The stress hyporesponsive period is essential for the adaptation to maternal–neonatal separation that normally occurs in rats (Calhoun, 1963). Our study *confirmed* that the adrenal stress hyporesponsive period occurs in PD7 and PD14 pups, which is most likely a result of decreased adrenal sensitivity to ACTH (Arai & Widmaier, 1993; Brake, Arai, As‐Sanie, Jefcoate, & Widmaier, 1999; Lee & Widmaier, 2005; Nagaya, Arai, & Widmaier, 1995; Zilz et al., 1999) and not explained by a change in corticosteroid‐binding globulin (CBG) (Johnson et al., 2013). We confirmed that the corticosterone hyporesponsive period in the neonatal rat occurs despite a large increase in ACTH, as we have shown previously (Bodager et al., 2014; Bruder et al., 2008; Chintamaneni et al., 2013). Other studies have also suggested that the stress hyporesponsive period is the result of a decrease in critical adrenal genes and factors in the neonatal steroidogenic pathway (Brake et al., 1999; Lee & Widmaier, 2005; Zilz et al., 1999).

Testosterone may lead to a change in plasma and intrapituitary CBG secondary to increased nuclear uptake, which could theoretically alter the HPA axis response by changing local concentrations of free corticosterone (Viau & Meaney, 2004; Viau, Sharma, & Meaney, 1996). This could in part explain the dose‐related effects of flutamide on the HPA axis response to acute, neonatal hypoxia. However, a change in plasma CBG does not explain the age‐related effects we demonstrated since plasma CBG increases from PD2 to PD7 and then to PD14 (Johnson et al., 2013).

### 4.4 Flutamide dosage

Previous studies have shown that AR antagonism is achieved at a flutamide dose of 10 mg/kg, while minimizing neonatal hepatotoxicity and male rat feminization (Gray et al., 2006; McCormick & Mahoney, 1999). Since there was concern that flutamide did not achieve significant antagonism at 10 mg/kg, we also evaluated a 5‐times higher dose (50 mg/kg). High‐dose flutamide seems to act like an agonist whereas adequate antagonism was achieved at 10 mg/kg. This was shown by suppression of the HPA‐axis with 50 mg/kg flutamide treatment and inhibition of negative feedback at 10 mg/kg. Therefore, we suggest that 50 mg/kg dosage was too high when studying the neonatal rat HPA axis. Additionally, we measured gonadotropins and gonadal steroids to assess AR effectiveness. In general, standard dose flutamide demonstrated the expected increase in male FSH and testosterone due to AR antagonism. High‐dose flutamide, however, resulted in suppression of the rat pituitary‐gonadal axis suggesting AR agonism. In fact, in the PD7 female, high‐dose flutamide suppressed FSH below baseline. This further shows that high‐dose flutamide was not an appropriate treatment and that standard dose flutamide is an appropriate dosage regarding receptor antagonism.

### 4.5 The effect of flutamide on granulosa cell function

AR expression in the rat granulosa cell has previously been described, but its physiological significance has not been firmly established (Daniel & Armstrong, 1986; Hillier & De Zwart, 1981; Nimrod, Tsafriri, & Lindner, 1977; Tetsuka & Hillier, 1996). Previous studies have suggested that androgen can function both as a substrate for estradiol as well as enhance follicular maturation by increasing FSH‐induced aromatase gene expression (Rifka et al., 1978). This action is mediated by the AR. Furthermore, excess androgen may actually induce granulosa cell apoptosis (Tetsuka & Hillier, 1996). Therefore, tight regulation of AR expression is necessary in female rat reproductive tissue.

In the PD21 normoxic female, high‐dose flutamide suppressed FSH and estradiol compared to the standard dose of flutamide. Interestingly, PD21 females treated with both standard and high‐dose flutamide exhibited an estradiol response to hypoxia. These findings may have occurred because of a direct interaction of flutamide with the rat granulosa cell. For example, high‐dose flutamide may have modulated the function of the AR in the neonatal ovary (Knapczyk‐Stwora et al., 2019). This was not observed with standard dose flutamide, since significant gonadal concentrations are not achieved at 10 mg/kg, as described previously. The increase in estradiol observed under hypoxia occurred independently of FSH. One explanation could be that hypoxia induces an intraovarian effect only in the presence of flutamide. Hypoxia has been shown to upregulate vascular endothelial growth factor (VEGF) in the swine granulosa cell (Basini et al., 2004). VEGF induces expression of 17ß‐estradiol in mice granulosa cells and is a key mediator of follicle maturation (Sargent et al., 2015). Therefore, it is possible that our observation of increased estradiol in hypoxic rats is a result of increased VEGF or another similar factor, occurring independently of a rise in FSH. The direct mechanism regarding hypoxia‐induced increases in estrogen in the neonatal rat is a potential area of further study.

### 4.6 Perspectives

We conclude that the differential, short‐term effects of flutamide in the first 3 weeks of neonatal life renders it a less than optimal agent to reliably block the androgen effects in the newborn in order to evaluate androgen‐induced programming of subsequent sexually dimorphic adult behavior (Bingham, Gray, et al., 2011; McCormick et al., 1998; McCormick & Mahoney, 1999; Ongaro et al., 2011; Vega Matuszczyk et al., 1990). However, it does reveal possible unique androgen receptor‐mediated control of neonatal endocrine function possibly related to the effects of the androgen receptor in the hypothalamus, pituitary, adrenal, and/or gonads. For example, the effect of FSH on granulosa cell development may be altered by local androgen effects independently of changes in circulating gonadotropins (Tetsuka & Hillier, 1996). It may also have off target effects in the neonate not found in the adult. It is also clear that the dose of flutamide one chooses to use in the neonate is critical. We chose the “standard” dose because it does not cause major changes in early neonatal sexual development and clearly, higher neonatal doses have untoward effects even in the neonate (Gray et al., 2006; McCormick & Mahoney, 1999). The study of short‐ and long‐term roles of gonadal steroids on subsequent sexual dimorphisms in adult gene expression and phenotype continues to be an important area of study requiring complementary, validated experimental approaches.

## CONFLICT OF INTEREST

The authors have nothing to disclose.
